# BRAF V600E and TERT Promoter Mutations in Papillary Thyroid Carcinoma in Chinese Patients

**DOI:** 10.1371/journal.pone.0153319

**Published:** 2016-04-11

**Authors:** Jian Sun, Jing Zhang, Junliang Lu, Jie Gao, Xinyu Ren, Lianghong Teng, Huanli Duan, Yansong Lin, Xiaoyi Li, Bo Zhang, Zhiyong Liang

**Affiliations:** 1 Department of Pathology, Chinese Academy of Medical Sciences and Peking Union Medical College Hospital, Beijing, People's Republic of China; 2 Department of Pathology, Xuanwu Hospital, Capital Medical University, Beijing, People's Republic of China; 3 Department of nuclear medicine, Chinese Academy of Medical Sciences and Peking Union Medical College Hospital, Beijing, People's Republic of China; 4 Department of surgery, Chinese Academy of Medical Sciences and Peking Union Medical College Hospital, Beijing, People's Republic of China; 5 Department of ultrasound, Chinese Academy of Medical Sciences and Peking Union Medical College Hospital, Beijing, People's Republic of China; Mayo Clinic, UNITED STATES

## Abstract

**Background:**

The BRAF V600E and telomerase reverse transcriptase (TERT) promoter mutations have been reported in papillary thyroid carcinoma (PTC). The aim of this retrospective cross-sectional study was to add further information regarding the prevalence of the BRAF V600E and TERT promoter mutations in Chinese PTC and their clinicopathological associations.

**Methods:**

We detected the BRAF V600E mutation and TERT promoter mutations in 455 Chinese PTC patients and analyzed the association of these mutations with several clinicopathological features.

**Results:**

The BRAF V600E mutation was detected in 343 (75.4%) of 455 cases and was significantly associated with older age (p<0.001) and conventional subtype (p = 0.003). TERT promoter mutations were detected in 19 (4.4%) of 434 PTCs and were associated with older age (p<0.001), larger tumor size (p = 0.024), and advanced TNM stage(p<0.001). Of the 19 patients that were positive for TERT promoter mutations, 18 (94.7%) also harbored the BRAF V600E mutation.

**Conclusion:**

We determined the prevalence and clinicopathological associations of BRAF V600E and TERT promoter mutations in Chinese PTC patients. TERT promoter mutations but not the BRAF V600E mutation were associated with more advanced TNM stage upon diagnosis.

## Introduction

Papillary thyroid carcinoma (PTC) is the most common endocrine malignancy and its incidence is rapidly increasing globally[1]. PTC is highly curable, and the overall survival rate is reported to be 90%. However, up to 10% of patients with PTC eventually die as a result of this disease[2]. Several clinical factors predict poor prognosis in patients with PTC: older age (>45 years), tumor size >5 cm, extrathyroidal extension, multifocal tumors, lymph node metastasis, distant metastasis, an aggressive histological subtype, advanced TNM stage, and recurrence[3].

Several genetic changes have been associated with PTC; the most common is the BRAF V600E mutation. A recent large-cohort, multicenter study identified an association of the BRAF V600E mutation with a higher mortality rate in patients with PTC[4]. However, several other studies have reported no or a partial association of the BRAF mutation with high-risk pathological characteristics[5–7]. The association of the BRAF V600E mutation with more aggressive clinicopathological features in patients with PTC thus remains controversial.

Telomerase reverse transcriptase (TERT) is the catalytic subunit of telomerase, which plays a key role in cellular immortality[8]. Two TERT promoter mutations (1 295 228 C>T and 1 295 250 C>T, referred to as124C>T and146C>T, respectively, in the following text), particularly the 124C>T mutation, have been identified in bladder cancer and glioblastoma[9], suggesting a role for TERT promoter mutations inhuman tumorigenesis. Recent studies have reported TERT promoter mutations in thyroid cancers; these mutations are particularly prevalent in aggressive thyroid cancers and BRAF mutation-positive PTC[10,11]. Another study observed that TERT-mutant tumors are associated with a significantly higher prevalence of distant metastasis and poorer survival, regardless of BRAF status[12].

In this single-center, retrospective study, we evaluated the prevalence of the BRAFV600E mutation and TERT promoter mutations in Chinese patients with PTC and the relationship between these genetic mutations and various clinicopathological features.

## Materials and Methods

Patients who were diagnosed with primary PTC and underwent radical resection at Peking Union Medical College Hospital between January 2010 and December 2012 were enrolled in the present study. The following exclusion criteria were applied: less than subtotal thyroidectomy, unavailability of formalin-fixed paraffin-embedded (FFPE) tissue blocks, and insufficient clinical information. Archived hematoxylin and eosin-stained slides were reviewed by two experienced pathologists to confirm the pathological diagnosis and to obtain detailed pathological information, including tumor size, multifocality, histological subtypes or variants, and the presence of lymph node metastasis. Clinical information such as age, sex, and TNM staging of the tumor were retrieved from medical records. TNM staging was determined based on the 7^th^ edition of the American Joint Committee on Cancer (AJCC)/Union for International Cancer Control (UICC) TNM classification system[13].

This study was conducted with the approval of the Ethics Committee of the Peking Union Medical College Hospital, and written informed consent was obtained from all patients. During data collection, all authors had access to identifying information regarding the patients. The study was conducted in accordance with the approved protocol.

### DNA extraction

DNA was extracted from tissue samples using the QIAGEN QIAamp DNA FFPE Tissue Kit (56404, QIAGEN) following the manufacturer’s protocol and in 50 μl of buffer ATE (included in the kit). The absorbance of the extracted DNA was measured using a Merinton SMA4000spectrophotometer (Merinton Inc., Beijing, China), and the DNA was diluted with distilled water to a final concentration of approximately 2–3 ng/μl.

### Detection of the BRAF V600E and TERT promoter mutations

We tested the BRAF V600E mutation using a China Food and Drug Administration (CFDA)-approved human BRAF V600E ARMS-PCR kit (Amoy Diagnostics Co. Ltd, Xiamen, China). The quality of the extracted DNA was verified by the amplification of a housekeeping gene, which was reported in the HEX channel. Amplification was performed using the following cycling conditions on an ABI Prism 7500 thermocycler (Life Technologies, Carlsbad, California, USA):95°C for 5 min; 15 cycles of 95°C for 25 s, 64°C for 20 s, 72°C for 20 s; and 31 cycles of 93°C for 25 s, 60°C for 35 s, 72°C for 20 s. The FAM and HEX signals were collected at 60°C. The run files were analyzed and interpreted as specified in the manufacturer’s manual.

We used classic Sanger sequencing to detect TERT promoter mutations. The TERT promoter region was amplified using the primers 5-AGTGGATTCGCGGGCACAGA-3 (sense) and 5-CAGCGCTGCCTGAAACTC-3 (antisense) and the PCR conditions previously described by Liu et al (11). The quality of the PCR products was assessed by gel electrophoresis, followed by purification and sequencing of the products on an ABIPRISM 3730XL automated genetic analyzer (Life Technologies, Carlsbad, California, USA).

### Statistical analysis

Clinicopathological information was summarized using descriptive statistics. Patient age was summarized as the mean±standard deviation (SD), and tumor size was summarized using median and quartiles. Categorical variables were analyzed using the chi-square test or Fisher’s exact test as appropriate. Patient age and tumor size were compared using independent *t*-tests and the Mann-Whitney *U*-test, respectively. Data analysis was performed using SPSS software version 17 (SPSS Inc., California, USA). Missing data were not included in the calculation, and p<0.05 (or p<0.01 when multiple comparison was applied to compare the mutation rate differences between histological subtypes) was considered statistically significant.

## Results

### Demographic information of enrolled patients

The present study enrolled 523 patients. After applying the exclusion criteria, the final sample size was 455 cases, which included 124 (27.3%) males and 331 (72.7%) females. The mean age of the patients was 41.1±11.80 years, with a range of 11 to 73 years. Among the patients,309 (67.9%), 102 (22.4%), 35 (7.7%) and 9 (2.0%) were classified as conventional, follicular, solid, and other subtypes, respectively. Clinical staging information was available for all 455 patients. Clinicopathological and molecular profile of each patient was supplied in S1 Table. The demographic information of enrolled patients is summarized on the basis of the BRAF mutation or TERT promoter mutations in Table 1.

**Table 1:** Clinicopathological associations of BRAF V600E and TERT promotermutations.

Parameter	BRAFV600E mutation(N = 455)		p Value	TERT promoter mutation(s) (N = 434)		p Value
	Positive	Negative		Positive	Negative	
**Number of patients**	343(75.4%)	112(24.6%)		19(4.4%)	415(95.6%)	
**Gender**			0.389			0.791
F	246(71.7%)	85(75.9%)		15(78.9%)	305(73.5%)	
M	97(28.3%)	27(24.1%)		4(21.1%)	110(26.5%)	
**Age(years)**			<0.001			<0.001
mean±SD	42.5±11.1	36.8±12.9		52.79±4.74	40.40±1.11	
95% Confidence Interval	41.32–43.67	34.33–39.19		47.70–57.87	39.29–41.51	
**Size(mm)**			0.272			0.024
Median quartiles	10.00(6.00–15.00)	10.50(6.25–15.75)		14.00(8.00–22.00)	10.00(6.00–15.00)	
**Multifocality**			0.717			0.302
single	242(75.9%)	77(68.8%)		11(57.9%)	294(70.8%)	
multifocal	101(74.3%)	35(31.3%)		8(42.1%)	121(29.2%)	
**Variants types**			0.001			0.022
Conventional	245 (71.4%)	64(57.1%)		13(68.4%)	282(68.0%)	
follicular	66 (19.2%)	36(32.1%)		1(5.3%)	97(23.4%)	
solid	27(7.9%)	8(7.1%)		4(21.1%)	29(7.0%)	
other	5(1.5%)	4(3.6%)		1(5.3%)	7(1.7%)	
**LN metastasis**			0.24			0.054
yes	261(76.1%)	79(79.5%)		18(94.7%)	309(74.5%)	
no	82(23.9%)	33(20.5%)		1(5.3%)	106(25.5%)	
**TNM stage**			0.09			<0.001
I-II	248(72.3%)	90(80.4%)		6(31.6%)	316(76.1%)	
III-IV	95(27.7%)	22(19.6%)		13(68.4%)	99(23.9%)	

### Association of the BRAF V600E mutation with the clinicopathological characteristics of PTC

BRAF genotyping was successful for all 455 cases. The BRAFV600Emutation was detected in 343 (75.4%) of 455 patients. The BRAF-positive patients were significantly older than the BRAF-negative patients (42.5 vs. 36.8 years, p<0.001). No significant relationship was observed with respect to gender (p = 0.389), tumor size (p = 0.387), multifocality (p = 0.717) or lymph node metastasis (p = 0.240). Multiple comparisons revealed significant differences in the prevalence of the BRAF V600E mutation between conventional PTC and the follicular variant of PTC (p = 0.003). Nine cases in our cohort were classified as “other” variants of PTC, including 4 patients with a tall-cell variant and 5 with a disseminated sclerosing variant. The BRAF V600E mutation was detected in 3 of 4 (75%) tall-cell variant PTCs and none of the disseminated sclerosing variant PTCs. Advanced TNM stage was more common among the BRAF-positive patients, with marginal significance(p = 0.090). Distant metastasis occurred in 3 patients with PTC and did not differ significantly between the BRAF-positive and BRAF-negative groups.

### Association of TERT promoter mutations with the clinicopathological characteristics of PTCs

TERT promoter mutation analysis was unsuccessful in 21 cases due to low nucleic acid quality. Of the remaining 434 cases, the mutation was detected in 19 (4.4%); 18 (94.7%) had the 124C>T mutation and 1(5.3%) had the 146C>T mutation. Compared to TERT promoter mutation-negative patients, TERT promoter mutation-positive patients tended to be older (52.8 vs. 40.4 years, p<0.001), and present with a larger tumor (14.0 vs. 10.0 mm, p = 0.024). The -124C>T mutation was detected in 13 of 295 (4.4%) conventional PTCs, 1 of 99 (1.0%) follicular PTCs, 3 of 33 (9.1%) solid subtype PTCs, 1 of 3 (33.3%) tall cell subtype PTCs, and 0 of 4 disseminated sclerosing subtype PTCs. The 146C>T mutation was detected only in one solid subtype case and not in the other PTC subtypes. Multiple comparisons revealed that TERT promoter mutations tended to occur more frequently in solid variants than in tall cell variants; however, the difference was only marginally significant (p = 0.014). TERT promoter mutations were also significantly associated with more advanced TNM stage (p<0.001). No significant relationship was observed with respect to gender (p = 0.791), multifocality (p = 0.826), or lymph node metastasis(p = 0.054).

### Coexistence of TERT promoter and BRAF V600E mutations and their clinicopathological significance

A TERTpromoter mutation was detected in 0.9%(1 of 106) of BRAF V600E mutation-negative PTCs vs.5.5% (18 of 328) of BRAF mutation-positive PTCs, revealing a tendency toward co-occurrence of these mutations (McNemar Test, p<0.001). Compared with the remaining cases (BRAF V600E mutation alone or BRAF wild type and TERT promoter wild type), the coexistence of BRAF V600E and TERT promoter mutation was associated with virtually all high-risk factors, such as older age (p<0.001), larger tumor(p = 0.025), lymph node metastasis(p = 0.040), and advanced TNM stage(p<0.001). In contrast to cases harboring only BRAF V600E mutations, simultaneous BRAF and TERT promoter mutations were associated with older age upon diagnosis (p<0.001), larger tumor size (p = 0.011), more advanced TNM stage (p<0.001), and the solid subtype of PTC in contrast to the follicular subtype (p = 0.006) (S2 Table). In contrast to BRAF wild type and TERT promoter wild type cases, simultaneous BRAF and TERT promoter mutations were associated with older age upon diagnosis (p<0.001), more advanced TNM stage (p<0.001), and the solid subtype of PTC in contrast to the follicular subtype (p = 0.003). Simultaneous mutations also displayed a trend of association with larger tumor size that was not statistically significant (p = 0.056) (S3 Table). In TERT promoter mutation-negative PTC cases, the BRAF V600E mutation was associated with older age (p<0.001) and conventional subtype (p = 0.007) but not with other clinicopathological features.

## Discussion

BRAF V600Eis the most common mutation in PTC and plays a role in the initiation of follicular cell transformation [14]. Attempts to associate this mutation with poor outcome or other predictors, such as larger tumor size, multifocal lesions, distant metastasis, and recurrence, have yielded conflicting results [7]. Consequently, investigators have sought possible molecular confounding factors. TERT promoter mutations have been reported in glioma, breast cancer, and bladder cancer [9, 15] and were recently identified in thyroid malignancies. In contrast to the BRAF V600E mutation, the association of TERT promoter mutations with poor prognosis is well established [10, 12, 16]. However, in studies examining both the BRAF and TERT promoter mutations, the true prognostic efficacy of the BRAF mutation for PTC patients and its potential confounding by TERT promoter mutations have remained undetermined [11, 17, 18].

In this retrospective, single-center study, we investigated BRAF V600E and TERT promoter mutational status in 455 PTC cases in China and attempted to associate these mutations with different clinicopathological features of PTC. The BRAF V600E mutation was associated with older age and conventional subtype but not with more well-established prognosticators such as tumor size, multifocality, or TNM stage. Our results are consistent with the results of a study by Trovisco et al that enrolled 280 European patients [19] but conflict with the results of Xing et al, who reported an association of the BRAF V600E mutation with higher mortality among PTC patients [4]. However, the BRAF V600E mutation plays a role in the lack of avidity of PTCs for radioactive iodine[20], thus explaining the higher recurrence rate[21] and increased risk of disease-specific death.

TERT promoter mutations were associated with older age, larger tumor size, the solid variant of PTC, and more advanced TNM stage in the present study. Our results suggest that TERT promoter mutations indicate a poorer prognosis, consistent with previous studies[11, 16, 22]. Of 19 TERT promoter mutation-positive cases, 18(94.7%)also harbored a BRAF V600E mutation. A similar association was reported by Xing et al.[17], who observed a lower rate of coexisting mutations. Such findings are important because in the presence of highly coexisting mutations, it is difficult to control for the effect of the BRAF mutation, and thus the true prognostic efficacy of TERT promoter mutations cannot be distinguished. However, after excluding the 19 TERT promoter mutant cases, the association between the BRAF V600E mutation and clinicopathological features remained unchanged. Thus, the BRAF V600E mutation is associated with older age and conventional PTC but not more established prognosticators such as tumor size, multifocality, or TNM stage in TERT promoter-negative cases. These results suggest that the TERT promoter mutations might be more significantly associated with established prognostic factors such as more advanced TNM stage compared to the BRAF mutation.

The prevalence of the BRAF V600E mutation in the present study was 75.5%, much higher than figures previously reported for Western populations. A recent study by Liu et al reported a prevalence of 61.3% for the BRAF V600E mutation in Chinese PTC patients who had been exposed to various levels of iodine intake[11]. These differences in prevalence might be the result of differences in methodology. The limit of detection (LOD) for mutant alleles was as low as 1% for the ARMS-PCR method used in the present study[23, 24]. By contrast, the LOD of Sanger sequencing is usually as high as 10%. Thus, low-frequency BRAF V600E mutations might have not been identified in previous studies.

In this study, the prevalence of TERT promoter mutations was 4.4% in patients with PTC, lower than in previous studies[10, 11, 17]. Interestingly, Liu et al reported an exceptionally higher prevalence of TERT promoter mutations in Chinese PTC patients compared to that reported in the present study (11.3% vs. 4.4%). Their study and ours both enrolled patients with similar age ranges and an identical proportion of advanced-stage patients and adopted Sanger sequencing for the analysis of the TERT promoter region. However, tumor size was significantly larger in their study than in the present study (3.14±1.62 cm vs. 1.61±0.97 cm for TERT mutation-positive cases and 2.48±1.58 cm vs. 1.14±0.72 cm for TERT mutation-negative cases in Liu’s and our studies, respectively). Consequently, the sample in the present study might represent PTC cases with smaller tumor size[11]. In both studies, TERT promoter mutation was associated with larger tumor size, potentially explaining the lower prevalence of TERT promoter mutations among our cases.

In addition to the relationship between small tumor size and a lower TERT promoter mutation rate, the small tumor sizes in the present study also indicate that our sample primarily included low-risk PTCs. This characterization is important because the exceptionally high BRAF V600E mutation rate in low-risk PTC cases may further support a lack of association between this mutation and the aggressiveness of PTC.

This study is subject to the following limitations. First, follow-up results were not provided, making the study less informative. Follow-up results were not reported because distant metastasis and disease-specific death were rare among the enrolled patients throughout the follow-up period, most likely due to an insufficient duration of follow-up and the low-risk characteristic of the patients. Second, the TERT promoter region was examined using Sanger sequencing. Although Sanger sequencing is considered the gold standard for genetic analysis, it suffers from a relatively high LOD. The sensitivity of this method only permits the identification of mutant alleles with a penetration of greater than 10%. By contrast, the analysis of BRAF V600E mutation status was performed using an ARMS-PCR kit, which permits the identification of mutant alleles with a penetration of 1% in a specimen. Thus, while we might have failed to detect some low-frequency TERT promoter mutations in our cohort, too many cases might have been categorized as BRAF V600E-positive in the present study. This conclusion does not necessarily imply that we introduced false-positives in the detection of BRAF V600E mutations but rather that some very low-penetration cases might have been considered BRAF V600E-positive cases in the statistical analysis. The association of such a low penetration of a mutant allele with poor prognosis remains largely unknown. While it is unclear how this inconsistent performance of the methodology affected the results, it might have contributed to the non-association between BRAF V600E and established prognosticators. Thus, future studies should examine not only the presence of the mutation but also the frequency of the mutant alleles to determine the genotype-phenotype association within the study cohort. Third, as a single-center study enrolling mostly low-risk PTC patients, the generalizability of the conclusions is limited to low-risk PTC patients from the northern part of China. Given these limitations, we were unable to draw definitive conclusions regarding the prognostic values of the BRAF V600E mutation and TERT promoter mutations. We will continue to follow these patients and perform re-analyses of the two targets using the same methodology used in the present study.

In conclusion, we have reported the prevalence and clinicopathological associations of BRAF V600E and TERT promoter mutations in Chinese PTC patients. These results suggest that TERT promoter mutations rather than the BRAF V600E mutation are associated with more advanced TNM stage upon diagnosis.

## Supporting Information

S1 Table Clinicopathological and molecular profile of each patient (n = 455). (DOCX)

S2 Table Clinicopathological significance of coexisting BRAF and TERT promoter mutations compared to single BRAF mutation. (DOCX)

S3 Table Clinicopathological significance of coexisting BRAF and TERT promoter mutations compared to BRAF and TERT promoter wild type. (DOCX)

## References

[1] Pellegriti G, Frasca F, Regalbuto C, Squatrito S, Vigneri R. Worldwide increasing incidence of thyroid cancer: update on epidemiology and risk factors. Journal of cancer epidemiology. 2013;2013:965212 10.1155/2013/965212 23737785 PMC3664492

[2] Hughes DT, Haymart MR, Miller BS, Gauger PG, Doherty GM. The most commonly occurring papillary thyroid cancer in the United States is now a microcarcinoma in a patient older than 45 years. Thyroid: official journal of the American Thyroid Association. 2011;21(3):231–6. 21268762 10.1089/thy.2010.0137

[3] Dean DS, Hay ID. Prognostic indicators in differentiated thyroid carcinoma. Cancer control: journal of the Moffitt Cancer Center. 2000;7(3):229–39. 10832109 10.1177/107327480000700302

[4] Xing M, Alzahrani AS, Carson KA, Viola D, Elisei R, Bendlova B, et al Association between BRAF V600E mutation and mortality in patients with papillary thyroid cancer. Jama. 2013;309(14):1493–501. 10.1001/jama.2013.3190 23571588 PMC3791140

[5] Fugazzola L, Mannavola D, Cirello V, Vannucchi G, Muzza M, Vicentini L, et al BRAF mutations in an Italian cohort of thyroid cancers. Clinical endocrinology. 2004;61(2):239–43. 15272920 10.1111/j.1365-2265.2004.02089.x

[6] Lee JH, Lee ES, Kim YS. Clinicopathologic significance of BRAF V600E mutation in papillary carcinomas of the thyroid: a meta-analysis. Cancer. 2007;110(1):38–46. 17520704 10.1002/cncr.22754

[7] Li C, Lee KC, Schneider EB, Zeiger MA. BRAF V600E mutation and its association with clinicopathological features of papillary thyroid cancer: a meta-analysis. The Journal of clinical endocrinology and metabolism. 2012;97(12):4559–70. 10.1210/jc.2012-2104 23055546 PMC3513529

[8] Blasco MA. Telomeres and human disease: ageing, cancer and beyond. Nature reviews Genetics. 2005;6(8):611–22. 16136653 10.1038/nrg1656

[9] Liu X, Wu G, Shan Y, Hartmann C, von Deimling A, Xing M. Highly prevalent TERT promoter mutations in bladder cancer and glioblastoma. Cell cycle (Georgetown, Tex). 2013;12(10):1637–8. 10.4161/cc.24662 PMC3680543 23603989

[10] Liu X, Bishop J, Shan Y, Pai S, Liu D, Murugan AK, et al Highly prevalent TERT promoter mutations in aggressive thyroid cancers. Endocrine-related cancer. 2013;20(4):603–10. 10.1530/ERC-13-0210 23766237 PMC3782569

[11] Liu X, Qu S, Liu R, Sheng C, Shi X, Zhu G, et al TERT promoter mutations and their association with BRAF V600E mutation and aggressive clinicopathological characteristics of thyroid cancer. The Journal of clinical endocrinology and metabolism. 2014;99(6):E1130–6. 10.1210/jc.2013-4048 24617711 PMC4037723

[12] Melo M, da Rocha AG, Vinagre J, Batista R, Peixoto J, Tavares C, et al TERT promoter mutations are a major indicator of poor outcome in differentiated thyroid carcinomas. The Journal of clinical endocrinology and metabolism. 2014;99(5):E754–65. 10.1210/jc.2013-3734 24476079 PMC4191548

[13] Edge Stephen DRB, Compton Carolyn C., Fritz April G., Greene Frederick L., Trotti Andrew. AJCC Cancer Staging Manual. 7 ed: Springer-Verlag New York; 2010 XV, 648 p.

[14] Caronia LM, Phay JE, Shah MH. Role of BRAF in thyroid oncogenesis. Clinical cancer research: an official journal of the American Association for Cancer Research. 2011;17(24):7511–7. 21900390 10.1158/1078-0432.CCR-11-1155

[15] Killela PJ, Reitman ZJ, Jiao Y, Bettegowda C, Agrawal N, Diaz LA Jr, et al TERT promoter mutations occur frequently in gliomas and a subset of tumors derived from cells with low rates of self-renewal. Proceedings of the National Academy of Sciences of the United States of America. 2013;110(15):6021–6. 10.1073/pnas.1303607110 23530248 PMC3625331

[16] Gandolfi G, Ragazzi M, Frasoldati A, Piana S, Ciarrocchi A, Sancisi V. TERT promoter mutations are associated with distant metastases in papillary thyroid carcinoma. European journal of endocrinology / European Federation of Endocrine Societies. 2015;172(4):403–13. 10.1530/EJE-14-0837 25583906

[17] Xing M, Liu R, Liu X, Murugan AK, Zhu G, Zeiger MA, et al BRAF V600E and TERT promoter mutations cooperatively identify the most aggressive papillary thyroid cancer with highest recurrence. Journal of clinical oncology: official journal of the American Society of Clinical Oncology. 2014;32(25):2718–26. 25024077 10.1200/JCO.2014.55.5094 PMC4145183

[18] George JR, Henderson YC, Williams MD, Roberts DB, Hei H, Lai SY, et al Association of TERT Promoter Mutation, but Not BRAF Mutation, with Increased Mortality in PTC. The Journal of clinical endocrinology and metabolism. 2015:jc20152690. 10.1210/jc.2015-2690 PMC4667158 26461266

[19] Trovisco V, Soares P, Preto A, de Castro IV, Lima J, Castro P, et al Type and prevalence of BRAF mutations are closely associated with papillary thyroid carcinoma histotype and patients' age but not with tumour aggressiveness. Virchows Archiv: an international journal of pathology. 2005;446(6):589–95. 15902486 10.1007/s00428-005-1236-0

[20] Lakshmanan A, Scarberry D, Green JA, Zhang X, Selmi-Ruby S, Jhiang SM. Modulation of thyroidal radioiodide uptake by oncological pipeline inhibitors and Apigenin. Oncotarget. 2015;6(31):31792–804. 10.18632/oncotarget.5172 26397139 PMC4741640

[21] Howell GM, Carty SE, Armstrong MJ, Lebeau SO, Hodak SP, Coyne C, et al Both BRAF V600E mutation and older age (>/ = 65 years) are associated with recurrent papillary thyroid cancer. Annals of surgical oncology. 2011;18(13):3566–71. 10.1245/s10434-011-1781-5 21594703

[22] Shi X, Liu R, Qu S, Zhu G, Bishop J, Liu X, et al Association of TERT promoter mutation 1,295,228 C>T with BRAF V600E mutation, older patient age, and distant metastasis in anaplastic thyroid cancer. The Journal of clinical endocrinology and metabolism. 2015;100(4):E632–7. 10.1210/jc.2014-3606 25584719 PMC4399285

[23] Ellison G, Donald E, McWalter G, Knight L, Fletcher L, Sherwood J, et al A comparison of ARMS and DNA sequencing for mutation analysis in clinical biopsy samples. Journal of experimental & clinical cancer research: CR. 2010;29:132. 20925915 10.1186/1756-9966-29-132 PMC2988723

[24] Huang T, Zhuge J, Zhang WW. Sensitive detection of BRAF V600E mutation by Amplification Refractory Mutation System (ARMS)-PCR. Biomarker research. 2013;1(1):3 10.1186/2050-7771-1-3 24252159 PMC3776245

