# Supplementary material for: BRAF V600E and TERT Promoter Mutations in Papillary Thyroid Carcinoma in Chinese Patients
Source: PLoS One. 2016 Apr 11;11(4):e0153319. doi: 10.1371/journal.pone.0153319 (PMC4827831; doi:10.1371/journal.pone.0153319)
Supplement: S2 Table — (DOCX) [file pone.0153319.s002.docx]

**S2 Table. Clinicopathological significance of coexisting BRAF and TERT promoter mutations compared to single BRAF mutation.**

|  | **BRAF mutation only** | **BRAF mutant and TERT mutant** | **p** |
| --- | --- | --- | --- |
| **Female** | 232(71.4%) | 14(77.8%) | 0.558 |
| **Age (years)** | 41.94±10.802 | 52.50±10.777 | <0.001 |
| **Tumor size (in mm, median and quartiles)** | 10 (6.00-15.00) | 14.5 (9.25-22.50) | 0.011 |
| **Multifocal** | 93(28.6%) | 8(44.4%) | 0.152 |
| **TNM(I-II)** | 243(74.8%) | 5(27.8%) | <0.001 |
| **TNM(III-IV)** | 82(25.2%) | 13(72.2%) |  |
| **LNM** | 244(75.1%) | 17(94.4%) | 0.085 |
| **Conventional** | 232(71.4%) | 13(72.2%) | 0.008 |
| **Follicular** | 66(20.3%) | 0(0.0%) |  |
| **Solid** | 23(7.1%) | 4(22.2%) |  |
| **Others** | 4(1.2%) | 1(5.6%) |  |

In contrast to BRAF-mutant-only cases, dual BRAF and TERT promoter mutations are associated with older age upon diagnosis, larger tumor size, more advanced TNM stages, and Solid subtype of PTC in contrast to follicular subtype.TNM, Tumor, Node and Metastasis; LNM, lymph node metastasis.
