# Supplementary material for: BRAF V600E and TERT Promoter Mutations in Papillary Thyroid Carcinoma in Chinese Patients
Source: PLoS One. 2016 Apr 11;11(4):e0153319. doi: 10.1371/journal.pone.0153319 (PMC4827831; doi:10.1371/journal.pone.0153319)
Supplement: S3 Table — (DOCX) [file pone.0153319.s003.docx]

**S3 Table.Clinicopathological significance of coexisting BRAF and TERT promoter mutations compared to BRAF and TERT promoter wild type.**

|  | **BRAF wt and TERT wt** | **BRAF mutant and TERT mutant** | **P** |
| --- | --- | --- | --- |
| **Female** | 85(75.9%) | 14(77.8%) | 1 |
| **Age (years)** | 36.76±12.982 | 52.50±10.777 | <0.001 |
| **Tumor size(in mm, median and quartiles)** | 10.5(6.25-15.75) | 14.5(9.25-22.50) | 0.056 |
| **multifocal** | 35(31.2%) | 8(44.4%) | 0.269 |
| **TNM(I-II)** | 90(80.4%) | 5(27.8%) | <0.001 |
| **TNM(III-IV)** | 22(19.6%) | 13(72.2%) |  |
| **LNM** | 79(70.5%) | 17(94.4%) | 0.041 |
| **Conventional** | 63(57.1%) | 13(72.2%) | 0.004 |
| **Follicular** | 36(32.1%) | 0(0.0%) |  |
| **Solid** | 8(7.1%) | 4(22.2%) |  |
| **Others** | 4(3.6%) | 1(5.6%) |  |

In contrast to BRAF wt and TERT promoter wt cases, dual BRAF and TERT promoter mutations are associated with older age upon diagnosis, more advanced TNM stages, and Solid subtype of PTC in contrast to follicular subtype. Dual mutations also show a trend of association with larger tumor size.TNM, Tumor, Node and Metastasis; LNM, lymph node metastasis.
